# Combination of Milk and Plant Proteins to Develop Novel Food Systems: What Are the Limits?

**DOI:** 10.3390/foods12122385

**Published:** 2023-06-15

**Authors:** Luis Gustavo Lima Nascimento, Davide Odelli, Antônio Fernandes de Carvalho, Evandro Martins, Guillaume Delaplace, Paulo Peres de sá Peixoto Júnior, Naaman Francisco Nogueira Silva, Federico Casanova

**Affiliations:** 1 Departamento de Tecnologia de Alimentos, Universidade Federal de Viçosa (UFV), Viçosa 36570-900, MG, Brazil; luisgusta.ln@gmail.com (L.G.L.N.); davide.odelli@ufv.br (D.O.); evandromartins@ufv.br (E.M.); 2 Laboratoire de Processus aux Interfaces et Hygiène des Matériaux, INRAE, 59009 Lille, France; guillaume.delaplace@inrae.fr (G.D.); paulo.peres-de-sa-peixoto-junior@inrae.fr (P.P.d.s.P.J.); 3 Center of Natural Sciences, Federal University of São Carlos (UFSCar), Campus de Lagoa do Sino, Buri 18290-000, SP, Brazil; naaman.nogueira@ufscar.br; 4 Research Group for Food Production Engineering, National Food Institute, Technical University of Denmark, Søltofts Plads, 2800 Kongens Lyngby, Denmark

**Keywords:** milk proteins, plant proteins, mixed systems, colloidal properties, innovative foods, sensorial properties

## Abstract

In the context of a diet transition from animal protein to plant protein, both for sustainable and healthy scopes, innovative plant-based foods are being developing. A combination with milk proteins has been proposed as a strategy to overcome the scarce functional and sensorial properties of plant proteins. Based on this mixture were designed several colloidal systems such as suspensions, gels, emulsions, and foams which can be found in many food products. This review aims to give profound scientific insights on the challenges and opportunities of developing such binary systems which could soon open a new market category in the food industry. The recent trends in the formulation of each colloidal system, as well as their limits and advantages are here considered. Lastly, new approaches to improve the coexistence of both milk and plant proteins and how they affect the sensorial profile of food products are discussed.

## 1. Introduction

The human population is continuously growing, and it is estimated to reach 9.7 billion people in 2050, which will naturally increase the demand for animal protein for human nourishment (United Nations, 2015). A report conducted by Poore and Nemecek (2018) [1] considered the environmental footprint of the production of 90% of global proteins based on land use, freshwater usage, GHG emissions, and chemical emissions in soil and water. The authors showed that proteins from animal sources (meat, dairy, eggs, and aquaculture) use ~83% of the world’s available farmland and are responsible for 56–58% of general emissions, providing, in the end, only 37% of food protein supply [1]. Thus, considering the crescent demand for proteins and the deployment of the Glasgow Climate Pact (UNFCCC, 2021), the development of sustainable production systems to obtain alternative protein sources is required.

Inside this scenario, plant proteins are good candidates to partially substitute animal proteins in food since their production process has been associated with low cost and low greenhouse effect [2]. Beyond this, plant proteins are less allergenic than animal counterparts [3]. The consumer’s increasing awareness of healthy and sustainable food products has recently enhanced the demand for plant-based proteins as food ingredients worldwide, and only in the United States, 83% of North American consumers are adding plant-based foods into their diets to improve health (NDC, 2019). Proteins from various vegetables have already been studied and employed as animal protein replacers in meat and dairy analog products [4,5,6].

Many researchers have highlighted the positive nutritional aspects of this kind of protein which, among others, include a reduced glycemic index, reduced incidence/probability of developing cardiovascular diseases, obesity, and metabolic syndromes; conditions that reduce the overall all-cause mortality [7,8,9]. Therefore, the incorporation of plant proteins is not only a necessity but also a consumer tendency to maintain its well-being and healthy condition [10].

Despite the advantages in the use of plant proteins for human nutrition, their pronounced taste and poor solubility limit their applicability in the food industry [11]. To overcome this techno-functional drawback, association with animal proteins, such as milk proteins, can be an interesting strategy to increase the use of plant proteins with low compromising of food sensorial aspects. Among the potential animal proteins that can be combined with the plant ones, milk proteins stand out due to high productions, easy isolation and purification by membrane filtration systems, stability in the dry form, tecno-functionality in dairy and non-dairy products and good acceptability for consumers [12,13,14]. The milk and plant proteins association can improve the sensorial and nutritional aspects of foods, increase the intake of plant proteins in processed foods, reduce costs of ingredients, decrease phase separation and/or syneresis in dairy gels [15,16]. It is desired that plant proteins addition in milk-based foods can improve some properties of the system, however, this addition can alter significatively the characteristics of products, which could result in consumer rejection.

Thus, the impact of this association as well as the optimization of protein interactions must be better understood for the development of innovative products with sensory characteristics suited to the needs of consumers. In recent years, consistent research has been delivered to study these associations in different colloidal states such as dispersions, foams, gels and emulsions [17]. Indeed, these interactions depend on several aspects such as type of proteins, protein ratio, pH, ionic strength, presence of salts; additionally, industrial processes such as temperature, acids and enzymes can cause protein modification [18].

In this context, this review aims to describe the scientific advances regarding how the mixing of milk and plant protein change the features of protein systems and how these new characteristics can be useful in the formulation of foods with new textural and sensorial aspects. Moreover, innovative approaches to modify protein techno-functionality will be discussed here as a possible way to improve this combined system, limiting their drawbacks and promoting their application in the food industry.

## 2. Milk Proteins

The main milk components are listed in Table 1 [19]. The raw fluid milk can be transformed into a variety of food products such as ice cream, concentrated milk, milk powders, yogurt, cheese, etc. These transformations come mainly from manipulating the structure and organization of milk proteins, which influence taste, appearance, texture, color, and stability of these products [20]. The milk protein fraction can be grouped into two main classes: caseins, which are thermal resistant and have an isoelectric point in pH around 4.6, and whey proteins, which are soluble at their isoelectric point (~pH 4.8–5.0) but are precipitated by increasing the ionic strength and temperature [21,22].

The structures and functional properties of these two main groups of milk protein will be better discussed in the following paragraphs.

### 2.1. Caseins

**Figure 1:**
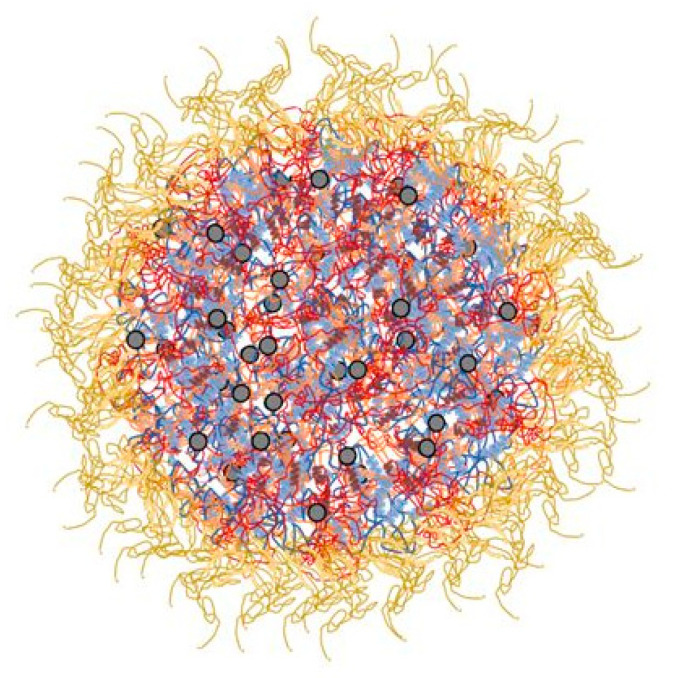
Casein micelle structure and its components: κ-casein (**yellow**); α-casein (**blue**); β-casein (**red**); calcium phosphate nanoclusters (**grey dots**).

Caseins compose about 80% of total milk proteins and are represented by four main fractions: αs1-, αs2-, β-, and k- caseins in a molar ratio of 11:3:10:4, respectively [20]. In natural milk conditions, these fractions interact with each other by hydrophobic and electrostatic interactions, and calcium phosphate nanoclusters forming supramolecular structures named casein micelles (CMs) (Figure 1). κ-casein fraction contributes to the electrostatic and steric repulsion among CMs and is the main casein responsible for stabilizing and maintaining CMs in suspensions [23].

Commercially, the separation of caseins from other constituents of milk, occurs by isoelectric precipitation, ultra and microfiltration, and rennet coagulation [24,25]. Acid caseins can be obtained by adjusting milk pH to 4.6, after that, a centrifugation step can separate the fractions. Due to their spherical structure, with large particle sizes, caseins obtained by acidification are insoluble in water and generally requires neutralization for their solubilization [26]. To overcome this problem, in food formulation caseins are typically applied in the form of sodium or calcium caseinates. They are produced from CMs by the addition of NaOH or CaOH to skimmed milk. The resulting caseinates are more soluble and have better water holding capacity (WHC) compared to native CMs thanks to their non-spherical shape and improved hydration of the particles, which confer small particle sizes of about ~20 nm [27]. Caseins in different configurations, i.e., CMs, caseinates, acid caseins, and rennet casein (caseins enzymatically precipitated), can be incorporated as food ingredients in a variety of food products such as waffles, cake mixtures, bread, cream liqueurs, coffee whiteners, processed meat and fish products and also dairy products such as cheese analogs and ice cream yogurt, among others [25]. Despite their nutritional features, the main reasons for caseins applications are their suitable functional properties. Indeed, thanks to their exceptional surface activity, emulsifying and self-assembly properties, and gelation and water binding capacities, caseins and caseinates are largely employed in food products as emulsifiers and foaming agents, fat replacers, and texture and thickening improvers [28]. These properties derive from the ability of caseins to be modified and form different colloidal systems such as dispersions, emulsions, foams, and gels [29].

### 2.2. Whey Proteins

In the past, whey was considered a waste created by the cheese and caseins production but the panorama has changed since then, mainly due to the discovery of its nutritional and techno-functional properties, which boost whey applications in the food industry [30]. Whey proteins account for approximately 20% of milk proteins and are composed mainly of β-lactoglobulin (60% *w*/*w*) and α-lactalbumin (20% *w*/*w*) with lower contents of immunoglobulins (10% *w*/*w*), bovine serum albumin (3% *w*/*w*), and lactoferrin (<0.1% *w*/*w*) [31]. Contrarily to the caseins, whey proteins are globular proteins with well-defined secondary, tertiary, and quaternary structures that depend on medium conditions such as pH, ionic strength, and temperature, but can also be modified by different treatments such as pressure, ultrasounds, pulsed electric field, and enzymatic reactions [22]. When the whey proteins are heated above their denaturation temperature, the molecular structure is unfolded and the formation of new hydrophobic interactions, hydrogen bonds, and disulfide bounds is favored [32].

Whey proteins are usually obtained by ultra and microfiltration. This technology allows the use of low temperatures and the absence of chemicals or enzymes added to the milk, which results in the purification of whey proteins with similar structure to their natural conformation, thus without interfering with their physicochemical properties. In the food industry, the main products obtained from whey processing are whey protein concentrates (WPCs) and whey protein isolates (WPIs). Those products can be used as ingredients in food formulations due to their ability to strengthen food gels and/or stabilize emulsions and foams. Additionally, WPCs and WPIs can be directly consumed by the final consumer after powder resuspension, giving protein solubility a paramount role for consumer acceptance [30].

## 3. Plant Proteins

### 3.1. Sources

**Figure 2:**
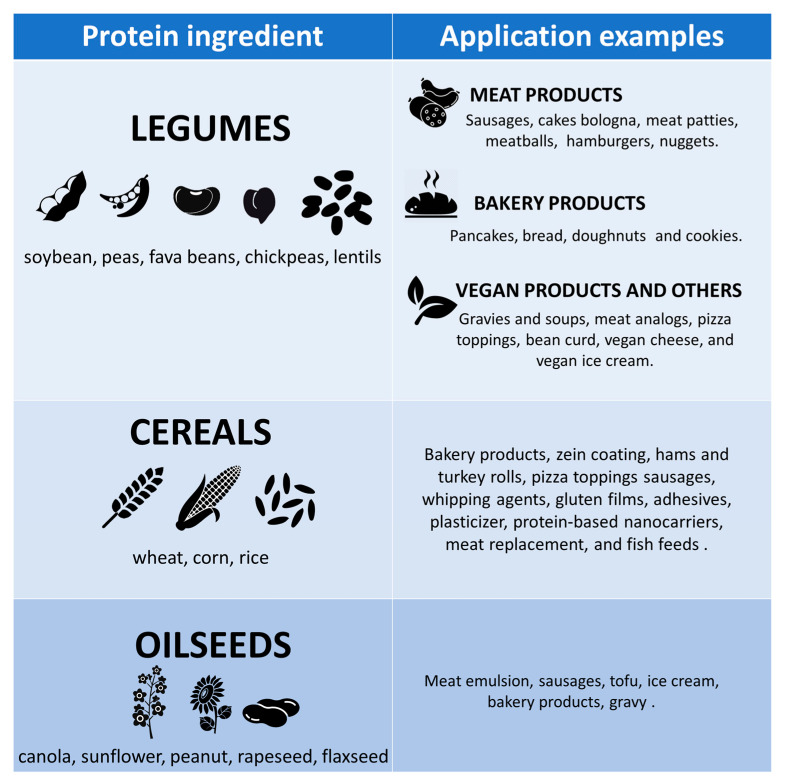
Main plant-based sources and some of their applications. Adapted from Akharume et al., (2021) [43].

Plant proteins are characterized by a different structure and morphology than animal proteins, which highly influences their functionality [33]. During their evolution history, plants have developed the ability to biosynthesize a large number of proteins for different purposes and can be generally classified into two different groups: “metabolic” and “storage” proteins. The first ones represent crucial proteins for the development of the plant, while the second ones consist of the reservoir of vital amino acids to sustain plant life [34]. These groups represent an important nutritional source for both humans and livestock or animal feed thanks to the presence of essential amino acids which can satisfy their nutritional requirements [35,36]. Plant proteins are generally obtained by dry or wet extraction methods as co- or by-products from various starting materials of the oil and starch extraction industries. More than 30 plant protein sources are currently used in food formulation, and overall, they can be organized into three general groups: legumes, cereals, and oilseeds [33] (Figure 2). Among the legumes, soybean and green peas are the most employed nowadays, but also proteins from other beans such as fava beans, chickpeas, and lentils are commonly requested by the food industry [37,38]. Regarding the cereals group, the main sources of proteins are provided by wheat gluten, corn zein, and rice, while proteins from oilseeds are separated from the oil, starch, and fibers of products such as canola, sunflower, peanut, rapeseed, and flaxseed [39,40,41,42].

### 3.2. Structure and Functionality

Proteins’ polypeptide composition, in terms of amino acids and functional groups, and their rearranged spatial structure greatly influence their physicochemical properties and functionalities [44]. Plant proteins display a specific morphology when they are biosynthesized, which allows them to express their biological functions. The natural 3D structure is obtained through the folding and interaction of the protein-peptide chains, driven by several forces such as van der Waals and hydrophobic attractions [34]. Hydrogen bonds, disulfide bonds, electrostatic and steric attraction/repulsion, torsional angles, and solvent interactions also participate in the morphology of the amino acid chains within a protein, but the same interactions can also occur within different protein molecules. For this reason, it is reasonable to believe that proteins physiologically exist in different states, which can range from monomers to oligomers and, at a certain concentration, to assemblies and aggregates, all characterized by this kind of natural forces [15,45]. It is good to know that any kind of process applied to the raw vegetable material is possibly able to interfere with these forces and thus influence biomolecules’ native structures and functionalities.

For example, the employed extraction methodology, purification, and any other processing method can largely modify protein three-dimensional organization. It has been proven that proteins extracted from the same source with different methodology may present a greater functionality variation than proteins extracted from different sources with the same method [46]. For instance, with a dry extraction, proteins tend to maintain their native organization, while with a wet extraction, different solvents are adopted such as water or an alkali, acid, or a salt solution which interact with the native proteins causing a potential disruption and rearrangement of their structures [15]. Therefore, it is fundamental to adapt all of these processes to obtain the desired characteristics of isolated proteins and be able to design specific food products.

When added as functional ingredients, proteins exhibit many roles in food matrices influencing for example their texture and structure but also their organoleptic properties such as flavor, color, odor, and appearance. Indeed, thanks to their amphipathic nature they can interact with other macronutrients such as carbohydrates and fats but also with water and air, working as gelling and thickening agents, stabilizers of foams and emulsions, film-forming polymers, and binding agents for fat and water, which all together represent colloidal properties [43]. Moreover, they could also have biological properties exhibiting antimicrobial and antioxidant effects [11]. Some examples of plant protein sources and their functional utilization in food formulation are resumed in Table 2.

## 4. Protein-Protein Interactions to Modify Food Techno-Functional Properties and Colloidal Properties

Protein techno-functionality can be described as the protein behavior during food processing and in a food system, a behavior which is strictly based on protein physicochemical properties, without necessarily including its biological and nutritional activities [47]. For example, caseins’ biological function is not to make dairy products, but their colloidal properties are responsible for several interactions that play a fundamental role in cheese and yogurt manufacturing. Physicochemical interactions such as electrostatic attraction and repulsion, hydrogen bonds, hydrophobic interactions, and disulfide bonds affect protein colloidal properties. Thanks to these properties, proteins interact with each other and other ingredients in food formulations determining their overall structure and colloidal state. Colloidal systems such as dispersions, gels, emulsions, and foams containing proteins are, thus, extremely influenced by their techno-functional properties, which can further be modulated during food processing, when other ingredients are added into the formulation and/or physical, chemical, or enzymatic treatments are employed. As complex systems, foods are usually composed of more than one colloidal state; therefore, the knowledge of how proteins behave in each of these colloidal states is precious to design any food formulation and can be used as a tool to predict and tailor the final product features.

The following paragraphs will specifically focus on the protein-protein interactions between milk and plant proteins, describing their role and characteristic in any type of colloidal state, in order to obtain a general consciousness of their relationship for the development of innovative food systems.

### 4.1. Milk: Plant Proteins Dispersions

A dispersion is a colloidal system where a solid material is dispersed into a liquid, where the solid is the dispersed phase, and the liquid is the continuous phase [48]. Thus, the formulation of beverages arises as to the direct application of the knowledge gained in these studies. Additionally, dispersions must be made before other systems, i.e., gels, emulsions, and foams, and the type of interactions, as well as the dispersion properties as viscosity, particle sizes, and solubility, affect the final product [49]. By the dispersion’s definition, solubility is the most important factor and can be understood as the resultant of the protein-protein and protein-solvent interactions [50]. The challenge increases when a high percentage of protein dispersed is required, as observed in high-protein beverages, mainly designed for the market of sports drinks [51]. The solubility of plant proteins, in general, is lower than milk proteins and can even be worse when high temperatures are used for the protein extraction. In mixed systems, the presence of a different protein can impact the overall system solubility. Ben-Harb et al., 2018 (Ben-Harb et al., 2018), observed an antagonistic effect in the solubility of mixed pea/milk proteins, where the mixture of pea and milk proteins was less soluble compared to each protein individually. However, other treatments can improve the solubility of mixed systems as demonstrated by Wang et al., (2019) [52], with the application of a pH-cycle technique. By variation of the dispersion pH from 12.0 to 7.0, the authors observed an increase in proximately 30 times of rice protein solubility when it was associated with WPI (1:1) compared to the pure rice suspension. The main reason for the observed phenomenon was attributed to proteins complexation, driven mainly by the formation of hydrogen bonds. Using the same method, Wang et al., (2018) [53], observed an increase in proximately 52 times in the solubility of rice protein when combined with sodium caseinate in the ratio 1:0.01. In addition, the increase in sodium caseinate content up to a 1:1 ratio did not significantly change the solubility of the systems, while when it was reduced, a lower solubility of rice proteins was detected.

The viscosity and the particle size of the proteins in dispersion also change regarding protein combination ratios, which directly impact the process parameters. It was observed by Singh et al., (2019) [54], that the mixture of milk protein concentrate (MPC) and soy protein hydrolytes (SPH) resulted in dispersions with higher viscosities when compared with the systems formed only by one type of protein at equal protein concentrations. The coagulation time of the systems also was impacted, SPH does not coagulate when exposed to 145 ℃ for 15 min, and the MPC took 14 min to present the first sign of coagulation. After the mix, depending on the ratio, coagulation time decreased to less than 2 min. These results, which are summarized in Table 3, show that the general processing carried out in the food industry for systems with only one protein source cannot be directly applied to mixed systems.

Therefore, it can be said that, in general, the presence of a mixture of proteins in a dispersion may negatively affect their solubility due to complex intermolecular interaction between the proteins and the solvent, causing higher molecular aggregates and precipitates. However, different treatments could be employed in order to modify the component structure and improve its overall solubility. For example, physical treatments such as homogenization, ultrafiltration, or ultrasounds have been applied to affect plant proteins particle sizes and second structures, generally enhancing their solubility [55]. Saricaoglu (2020) [56], used high-pressure homogenization to significantly reduce particle size of lentil proteins and increase their solubility, as well as influence their rheological properties. In the same context, Wang et al., (2020) [57], employed an ultrasound-assisted extraction method for pea protein isolate, which resulted in a partial protein unfolding and smaller particle size that significantly improved their dispersion. Additionally, chemical modification, such as phosphorylation, enzymatic hydrolysis, and biopolymer complexation, have been applied to modify protein functional groups, structure and viscosity in order to improve the interaction with the solvent [58,59,60].

Thus, solubility, viscosity and particle size, and how they are affected by different treatments have to be considered when a mixed protein system is designed in order to evaluate its stability and sensorial characteristics.

### 4.2. Milk: Plant Proteins Gels

A gel can be defined as a colloidal system where long thread-like molecules cross-link, chemically or physically, and/or entangle to such an extent that a continuous three-dimensional network is formed [61]. In rheological studies, a system where the elastic modulus (G′) is higher than its viscous modulus (G″) is defined as a gel, thus resembling a solid-like material [62]. In protein systems, gelation properties depend on intrinsic and extrinsic factors, such as amino acid composition, presence of disulfide bonds, hydrophobic and electrostatic interactions, protein concentration, ionic strength, temperature, pressure, and pH [32]. Particularly in a mixed system, the type of proteins, their concentration, and ratios affect the final gel properties. For example, the minimal protein concentration to achieve thermal and acid gelation was determined for mixed pea and β-lactoglobulin systems [63]. In the pure systems, the minimal concentration required for thermal gelation was 7 and 5% for pea proteins and β-lactoglobulin, respectively. The mixed systems minimal thermal gelation varied according to the protein ratio, 5% being the least concentration between the mixed samples for 1:4 pea: β-lactoglobulin protein ratio. Smaller values were found by Wong et al., (2013) [64], where the least gelation concentrations diminished when different protein rates were mixed, i.e., 3% of total protein concentration is required to form whey and pea gels. However, because of the synergistic enhancement of 2:8 pea/whey, 2% of total protein was necessary for gelation to occur. Additionally, the methodology used to obtain the gel is responsible for protein structures modification and their intermolecular interactions which influence the final features of the gel product.

#### 4.2.1. Heat-Induced Milk: Plant Proteins Gels

Protein gelation can occur when a sufficient amount of energy, in the form of heat, is applied to a system. Generally, at high temperatures, globular proteins unfold, exposing their hydrophobic residues that are hidden in the natural conformation. Once exposed, the amino acids can associate by hydrophobic interaction, Van-der-walls forces, and hydrogen bonds or can associate more strongly with disulfide bonds [46]. These new interactions between the protein chains lead to aggregation and a three-dimensional structure starts to form. In milk processing, heat treatment is used to promote aggregation between whey proteins and CMs, which in turn leads to stiffer gels after acidification. Thus, when plant proteins are added to milk, the first question that appears is if plant proteins can aggregate with CMs as whey proteins. Some authors have investigated the interactions between CMs and pea and soy proteins after heat treatment [65,66,67,68].

The common approach to access this information is using small-amplitude oscillation shear (SAOS) rheology technique to follow G′ during heat application. Silva et al., (2018) [65] studied the gelation profile of suspensions composed of CMs alone or in the presence of whey, pea, or soy protein at pH 5.8. As expected, a reinforcement of CMs gels was observed in the presence of whey protein, which was attributed to their co-aggregation. However, no reinforcement of the gel was observed even at high temperatures for both pea and soy protein, which suggests the absence of co-aggregation. Additionally, the protein ratios, i.e., the proportion between CMs and plant proteins, or protein concentrations, did not lead to their co-aggregation. In mixed systems where both CMs and plant proteins can form gels, the gel features are driven mainly by the protein that is in high concentration and the presence of two independent three-dimensional structures leads to less stiff gels [66]. Indeed, Mession et al., (2017) [68], studied the aggregation patterns of CMs and two fractions of pea protein, i.e., legumin, and vicilin, at pH 7.2 using reducing and non-reducing electrophoresis, DSC, and liquid chromatography. They concluded that during heat treatment, denaturation of both pea protein fractions took place, followed by the formation of protein aggregates. This aggregation occurs differently in each protein fraction, with the formation of disulfide bonds for legumin and non-covalent interactions for vicilin. However, the CMs did not participate in aggregation.

Despite the absence of co-aggregation between CMs and pea and soy proteins, the presence of the plant proteins impacts the availability of free calcium in the mixed systems, which seems to increase the CMs gelation temperature (Tgel) [65]. Tgel is defined as the temperature where the sol-gel transition occurs, and in the case of CMs suspensions, it is affected by free calcium concentration in the medium [69]. As the temperature increases the calcium solubility decreases, which leads to calcium precipitation on the CMs surface. As consequence, CMs destabilization occurs and ultimately leads to aggregation [70]. Thus, the less calcium available to precipitate, the harder it will be for aggregation to occur. As observed by Silva et al., (2018), pea and soy protein can bind calcium from the medium, where soy proteins bind more calcium than pea proteins, which resulted in higher gelation temperature of CMs in the systems where soy proteins were present. Thus, the authors argued that these plant proteins work as a chelating agent in mixed systems, increasing the heat stability of mixed systems in comparison to the suspensions of pure/isolated/native CMs.

The studies of how the plant protein specifically interacts with CMs are important to understand the potential application of mixed systems in the food industry. Ben-Harb et al., (2018) [18], studied heat-induced gelation in mixed milk/pea suspensions at pH 6.33. They found that 14.8% (*w*/*w*) mixed systems gel in protein ratios of 1:1 showed G′ as high as pea protein alone, while the sample containing solely milk fractions formed a weak gel at 14.8% (*w*/*w*) and did not gel at 7.4% (*w*/*w*) concentration. The data indicate that pea proteins were responsible for gel structuration, since CMs do not form gels when heated at a pH as high as 6.33 [69]. Nevertheless, pea proteins could not be the unique responsible for gel structure since the mixed systems only with 7.4% (*w*/*w*) of pea protein showed gel stiffness as high as the 14.8% (*w*/*w*) pea gels. Thus, interactions between whey and pea proteins may take place. Indeed, Wong et al., (2013) [64], studied the gel formation achieved by heating pea and whey protein in different rations, concentrations, and pH values. The best synergistic enhancement in G′ was achieved by 16% (*w*/*w*) total protein concentration, 2:8 pea/whey ratio at pH 6.0. In general, small amounts of pea protein increased the gel stiffness, but it varies depending on pH and protein concentration. Each protein has its isoelectric point and solubility, thus a pH value that promotes a similar aggregation profile of both proteins leads to the formation of a more homogeneous network. Additionally, the decrease in the electrostatic repulsion caused by pHs close to the protein isoelectric point leads to an increase in protein-protein interaction [64].

The mechanism of the interaction between β-lactoglobulin and pea after heat treatment at pH 7.2 was hypothesized by Chihi et al., (2016) [71]. The authors suggested that the unfolding of both protein types after heating exposed thiol groups and previously buried hydrophobic groups. In this way, the proteins started to self-aggregate, and aggregations between β-lactoglobulin and legumin potentially occur by disulfide bonds. Then, those small protein aggregates interact mainly by hydrophobic and/or electrostatic interactions, which increase their sizes. Despite the differences between soy and pea proteins, it is reasonable to think that the interactions with whey proteins for both plant proteins are similar. For instance, the formation of disulfide bonds after heat treatment of 6% (*w*/*w*) soy-whey protein mixed system has been proposed by Roesh and Corredig, (2005) [72]. The authors showed that when high amounts of whey are present, the incorporation of soy proteins occurs, and the formed aggregate is composed of both proteins. However, the presence of low amounts of plant proteins also led to the formation of aggregates formed solely by whey proteins. These diverse profiles led to differences in the gel network, where the gels formed with higher amount of whey protein showed a more homogeneous network and higher G′. The same feature of mixed soy-whey protein gels was observed, even at 12 and 16% (*w*/*w*) total protein concentration. Thus, in mixed systems, the whey protein is responsible for gel formation, while soy proteins appear as filler material within the gel structure [73]. To resume, the incorporation of soy protein in whey gels decreases the G′ and changes the network structure. Additionally, the modeling the soy/whey protein ratio allows the creation of 16% (*w*/*w*) protein gels with the same strength of 6% (*w*/*w*) [73].

In conclusion, it can be said that mixed-system heat-induced gel properties mainly depend on intrinsic factors such as type of proteins, their concentration, and ratio, and on environmental factors such as pH, ionic strength, and temperature applied for gel formation. Thus, at an industrial level, the strict control of all of these parameters can allow the design of products with desired techno-functional properties. In particular, the employment of different protein fractions may allow to obtain heat induced gel with the advantage of using less quantity of dairy protein as well as increasing the employment and consumption of plant-based proteins, obtaining similar gel structures to heat induced gel made out of animal proteins only.

#### 4.2.2. Acid Induced Milk: Plant Proteins Gels

The acid gelation is induced by pH modification toward the isoelectric point of the proteins. During the pH decreasing of protein suspensions, the electrostatic and steric repulsion between the proteins is reduced, which causes approximation between them, formation of new interactions, aggregation, and ultimately the formation of a continuous three-dimensional network [74]. In milk, the solubilization of calcium phosphate cannot be neglected once it causes protein rearrangement of the gel matrix. Acid gelation is widely applied in the dairy industry, mainly in the production of fermented milks and cheeses to develop desirable textural properties [75].

In mixed protein systems, the difference in protein origins and properties interfere in gel formation during acidification. For example, the pH, where the gelation starts for each protein, impacts directly the structure of the gel network [76]. In the acid gelation of pea and milk proteins, Ben-Harb et al., (2018) [18], observed that pea proteins play a major role in the first stages of gel formation because they reach the isoelectric point at higher rates, due to their lower buffer capacity. Chihi et al., (2018) [63], showed that the rates of acidification were equal for single and mixed systems composed of β-lactoglobulin and pea protein at 4% protein concentration. According to the authors, pea protein gelation occurred after 24 min of acidification in pH 6.6, while β-lactoglobulin gelation occurred 58 min after acidification at pH 5.7 in single systems. Thus, the increase in β-lactoglobulin in the mixed systems resulted in a decrease in gelation pH. The same was observed for an acid gel formed by mixing soy and cow’s milk at 4.5% total protein [76]. Soy gelation pH is around 6.0, while milk did not form gels by acidification in pH higher than 5.6. Thus, a gel network formed by the mixture of soy and milk in pH above 5.6 will be composed only of soy protein. In addition, the presence of milk proteins interfered in the soy network formation. If a rennet treatment is applied, the milk gelation pH rises to around 6.1; in this way, the formed gel network counts either with soy protein or milk protein contributions. The gelation of both proteins occurring at the same time increased G′ and formed a more homogeneous network compared to cow’s milk not treated with chymosin. However, the gels formed with only cow’s milk or soy milk presented higher G′ compared to the mixed systems. It indicates that there is no co-aggregation of the proteins and a network formation interferes in the other [76].

The presence of plant proteins in dairy products requires the evaluation of the changes during the production process and the interferences caused by the presence of lactic acid bacteria (LABs). Yousseef et al., (2016) [77], developed pea-milk yogurts with several LABs. In those systems, altering the pea/milk protein ratio from 0:100 to 40:60 at 4.5% total protein led to faster gel formation and increased the product acidity. The same occurred with the addition of lentil flour [37]. This phenomenon was explained by the lower buffer capacity in the systems with less casein content. Another effect after increasing pea protein amount was the increase in gels syneresis, which was related to the differences in gel network formation. The presence of pea protein decreased the firmness of the mixed gels compared to milk gels. It was suggested that the pea proteins prevented the formation of most homogeneous casein networks, thus weakening the resulting gel. This behavior highlights the possibility to develop gelled products of similar firmness with higher protein content using vegetable proteins.

The supplementation of milk with milk protein powder to increase the solid content, aiming at the development of a more elastic gel, is usual in the dairy industry. The substitution of milk protein powder for lentil flour as a source of solids was evaluated by Zare et al., (2011) [78]. The syneresis of yogurts supplemented with 3% lentil flower was similar to the samples with 3% milk powder. However, the syneresis increased when a lower quantity of lentil flour was added (1 and 2%). The increase in protein content in the samples, with the addition of more solids, lead to more water retention in the gel matrix compared to control samples. After 28 days of storage, the samples containing lentil flour presented G′ comparable to the samples supplemented with milk proteins, showing the potential of replacement of milk proteins for plant proteins.

The formulation of an acid-induced gel system does not exclude the application of a pre-treatment before gelling. Indeed, thermal treatment of milk is generally applied before fermentation in yogurt production to increase the stiffness of the final product. Pre-treatments, such as heat, are useful in modifying the proteins and the types of interactions between them, changing the building blocks of the acid gel. These building blocks are the foundation of the gel and their size and organization can be modulated by modifying the processing parameters such as pH, protein ratio, and the order of heat treatment, i.e., heating proteins separated with posterior mixing or mix the proteins with posterior heating [68]. The effect of the pre-heat treatment in the gel composed of sodium caseinate (CasNa), an important milk ingredient used in the dairy industry with several applications, and soy proteins were studied by Martin et al., (2016) [79]. The pH of the suspension during heat impacted the acid gel structure. In general, the heat treatment in lower pHs lead to more fragile and coarse gels. Additionally, the addition of soy protein without any heat treatment resulted in a gel with a coarser microstructure. Concerning the processing order, the heat treatment of only soy protein with posterior mixing with CasNa lowered the mechanical properties of the gel in comparison with CasNa alone. However, mixing the proteins before heat treatment increased the gel’s mechanical properties to a value close to CasNa alone. Similar results were observed by Chihi et al., (2018) [63], studying mixed β-lactoglobulin: pea protein gels. The authors showed that when the proteins were heated separately and then mixed, the gels show a more open and disordered structure with lower WHC compared to the sample in which the proteins were heated together before acidification. However, in a study of casein-pea gels, Mession et al., (2017) [68], observed that heating the proteins separately with their post-treatment mixture produced more elastic gels, explaining that the type of pea protein fraction utilized represents a critical factor for the final gel stiffness.

Thus, since each plant protein seems to have different acid gelation properties influenced by the presence of several fractions, by the specific isoelectric point, and by the characteristic structure and functional groups of the molecule, a general rule for all mixed milk-plant protein systems cannot be established. In other words, the nature of the proteins involved changes completely the characteristics of the acid gel. Even though in fermented products, plant proteins addition fastened the gelation and increased the acidity, when pea, soy and lentils proteins were added into acid dairy gels, their presence sterically inhibited the formation of a strong network, resulting in an increased syneresis and decreased gel firmness. However, the employment of heat treatment before acidification has shown relatively positive effects on gelling properties, opening the possibility of the application of a preliminary treatment to enhance proteins functionality. In this context, up to date, there is a lack of comprehensive information on the effects of different protein modification approaches to improve acid composite milk: plant protein gel, which needs to be carefully addressed for future perspectives in the food industry.

#### 4.2.3. Gelation Induced by Other Methods

Other physical methods employed to modify protein conformation, and thus structuring food, is the use of enzymatic reactions and/or ultrasounds. While enzymatic gelation has been known for many years in the dairy industry, i.e., the use of rennet in cheese-making processes, the utilization of ultrasound treatment is increasing in the food industry as a way to develop products with new features. Opposing the results reported by McCann et al., (2018) [73], who used heat treatment, Cui et al., (2020) [80], developed a whey-soy-based gel with higher hardness compared to the gels produced from the sole protein sources. However, the authors used a combination of ultrasound treatment and transglutaminase enzyme (Tgase). While ultrasounds treatment is known to promote the exposure of hidden amino acid residues, Tgase can promote a cross-link reaction between them. The higher hardness was recorded when the system was sonicated for 45 min. The ultrasound treatment also influenced the water holding capacity (WHC) of all different systems, with or without protein combination, and, in particular, the maximum WHC was recorded at 30 min of ultrasound treatment, without differences between the mixed and separated systems. In this specific enzymatic gelation, the caseins are the main product responsible for gel formation since they are more susceptible to Tgase action. However, the mixed gels had a lower store modulus (G′) compared to pure milk gels, perhaps because the presence of a different protein fraction inhibited the action of the enzyme [80]. A similar study was reported by Ma et al., (2022) [81], where a combined treatment of ultrasounds and enzymatic hydrolysis was applied to develop soy protein isolate (SPI) gels cross-linked by transglutaminase. In this case, papain-mediated hydrolysis was also added as a pre-treatment, in order to obtain a pool of different and modified proteins and peptides, which, associated with the ultrasounds, facilitated the cross-linking action of transglutaminase. The treated SPI gel showed a more uniform and dense structure, with significantly improved gel strength and water-holding capacity when compared to the untreated SPI gel. The results obtained by these studies highlighted the possible synergistic effects of these treatments, which could thus represent an effective way of improving gelling properties also of combined protein systems in which dairy and plant proteins coexist.

Another effective method to improve the gelation process is to combine the proteins with biopolymers with gelling capacity. In particular, protein amyloid fibrils have recently gained popularity for their ability to reinforce hydrogels thanks to their specific structure and availability of functional groups [82]. Protein amyloid fibrils can be obtained from a wide range of food proteins, including dairy and plant proteins, by hydrolysis and unfolding mediated by thermal treatments in acidic environments [83]. For example, Khalesi et al., (2021) [84] designed a gel composed of whey protein isolate and their amyloid fibrils and discovered that it was a brilliant strategy to improve the gelling properties of proteins. Indeed, the newly composed gel showed an enhanced elastic modulus by approximately 11 times compared to the control gel. This method has proven to be useful also for plant proteins where Wu et al., (2022) [85], created amyloid from pea proteins to form an enhanced gel for lutein encapsulation with better stability against environmental stresses. Additionally, the study conducted by Ge et al., (2022) [86], used amyloid fibrils from panda bean to reinforce the gel structure of pea protein isolate gel. Even though the water holding capacity and secondary structure were not modified, the gel strength was significantly enhanced and intermolecular interactions such as hydrogen bonds and hydrophobic interactions increased with increased fibrils concentration. Thus, this approach could also be used in mixed dairy and plant proteins gel to modify their textural and rheological properties, opening the area for innovative research that could finally be employed by the food industry.

As a conclusion, Table 4 summarizes all of the features and characteristics that have been studied so far about mixed milk-plant gels. From all of these insights, it can be stated that knowing the characteristic techno-functional properties of all the protein fractions involved in a mixed system and how they behave in different environmental conditions (pH; temperature; ionic strength) may allow the development of innovative gel products such as yogurt, cheese analogues, and beverages characterized by appropriate textural and sensorial properties.

### 4.3. Mixed Milk: Plant Proteins Emulsions

Emulsions are colloidal systems formed by two immiscible liquids, where one liquid is scattered in small droplets, the dispersed phase, into the other liquid, the continuous phase. Naturally, these systems are unstable, and require molecules able to adsorb in the interphases to decrease the interfacial tension and increase their stability [88]. In foods, emulsion systems are usually represented by water dispersed in oil (*w*/*o* emulsion) or oil dispersed in water (*o*/*w* emulsion). Margarine and butters are practical examples of the former, while mayonnaise and creams of the latter [48]. Milk by itself is an emulsion, where the lipids are finely dispersed in the continuous water phase, and stabilized by phospholipids, CMs, and whey proteins [89].

The combination of sodium caseinate and soy proteins with 5% oil fraction at 2% protein concentration in a 1:1 ratio was performed by Ji et al., (2015) [90]. The emulsions showed an average droplet size of 250 nm and a zeta potential of −45 mV at pH of 6.8. This high zeta potential value associated with the small droplet’s sizes conferred remarkable stability to the emulsion. The long-term stability of the emulsions stabilized by mixed proteins was higher than that of single proteins. After two weeks at room temperature, the droplet’s sizes grow from 250 nm to more than 1100 nm for the single protein emulsion, while it did not change for mixed system. Similar results were found by Hinderink et al., (2019) [17], where emulsions stabilized by combination of pea/WPI and pea/CasNa presented better stability after 14 days storage compared to emulsions where only one kind of protein was present, showing the synergic effect of the protein blends in the emulsion stability. The mixed emulsions layer was denser than the single proteins, and it may be a reason for better emulsion stabilization, where the systems were mainly stabilized by steric repulsion [90]. In the mixed systems, both proteins are absorbed at the interfacial layer with low concentration of proteins in the aqueous phase. However, during the storage time, a displacement of interfacial proteins can occur, as observed by Hinderink et al., (2019) [17], where whey proteins could substitute pea proteins in the interface, as well as pea protein displaced CasNa but without stability loss.

Liang et al., (2016) [91], studied emulsions formed by mixing CMs, pea, soy, and whey protein with a protein total concentration of 10% *w*/*w*, which is high if compared to the concentration of emulsifiers generally used. CMs mixed with plant proteins showed lower droplet size compared to a combination of CM-whey. As a general consideration, the higher the amount of whey, the higher the droplet size. Concerning heat stability, the systems containing soy protein presented better results in comparison to the systems formed by pea and whey.

Le Roux et al., (2020) [92], tried to produce infant formulas with a partial substitution of dairy proteins by pea and faba proteins and compared their functional properties with a traditional reference made entirely by dairy proteins. They found that the plant-based products showed, in general, very similar physicochemical and functional properties to the fully dairy infant formula reference. In particular, when the powders were mixed with an oil component to produce an emulsion, all of the samples presented similar emulsion stability with equivalent free fat release, independently from the protein source. However, it was also seen that pea and faba proteins were difficult to disperse and created larger aggregates with higher particle sizes when the powders were reconstituted. Further analyses are therefore necessary to elucidate the protein functions in such emulsion system, as well as to find a solution for particle size reduction.

The emulsions can also work as a delivery system for sensible hydrophobic bioactive molecules, which can be applied in the fortification of foods. The mixed system CasNa/ Soy proteins showed better protection properties compared to single protein systems, showing retention of vitamin A around 93% after three months of storage [90]. This protection over Vitamin A is due mainly to two factors: i. Proteins light deviation which diminishes Vitamin A light exposure and ii. ability of protein to bind metals in the aqueous phase. Milk: plant protein blends were also used as emulsifiers in lycopene emulsions [93]. The blends containing whey-soy and whey-pea presented better emulsion stability than the proteins alone. However, an antagonistic effect was observed in the blends of CasNa and the plant fractions, which cause emulsion destabilization after 7 days of production, probably caused by competitive absorption at the oil-drop surface between CasNa and pea [93].

The process that milk undergoes to develop milk products changes the protein structure and interaction. The understanding of the different processes employed in the dairy and beverage industries for mixed systems is relevant to give a more concrete idea of their potential uses. In addition to temperature, homogenization plays an important role during milk and plant beverage processing. The impact of the homogenization order, i.e., homogenize cow’s milk with cream followed by soy milk addition, or homogenize soy milk with cream followed by cow’s milk addition or homogenize both kinds of milk together was studied by Grygorczyk et al., (2014) [87]. The homogenization order modulates which protein will be predominant in the fat globule interfaces. When soy milk is homogenized with milk cream in absence of cows’ milk, soy proteins are the major constituents in the fat globule interface. The same occurs when milk is homogenized in absence of soy milk. However, when both milks and cream are homogenized together, the fat globule interface is composed mainly of milk proteins. The homogenization process did not have an impact on the fat droplet’s sizes.

Based on the results of all of the studies here considered, which key aspects are summarized in Table 5, it can be definitely said that mixed systems of milk and plant proteins may represent a very useful tool to improve stability and techno-functionalities of many food emulsions such as beverages, salad dressings, desserts, and cheese analogues. Their synergic effect at the oil droplet interface indeed forms a dense protein layer and maintain a steady droplet size for long periods of time; a feature that allows the inhibition of creaming and sedimentation which cause emulsion breakdown and instability. Moreover, dairy and plant protein mix also manifested interesting results as bioactive compounds carrier. Their employment in emulsion food formulations therefore could not only reduce the use of additives and emulsifiers but could also be used to improve the transport of several bioactive compounds, producing foods characterized by high protein content and healthy claims. However, sensorial properties of the composed foods need to be evaluated in order to also promote consumer acceptance.

### 4.4. Mixed Milk: Plant Proteins Foams

Foams are described as mixed systems in which gas bubbles are uniformly dispersed in a continuous liquid or solid phase. In food products such as cake, meringue, bread, and whipped toppings, they are essential parts contributing to properties such as texture and palatability [94]. Foams are thermodynamically unstable since gravitational forces and colloidal activities can be responsible for bubble coalescence and disproportionation, destabilizing the overall system. To prevent their collapse, surface-active substances are needed to reduce the surface tension around each air bubble and inhibit their burst, enhancing foam stability. Thanks to their amphipathic properties, proteins can be adsorbed at the interface of the phases and form a viscoelastic film which physically entraps air bubbles. Because of their high efficiency in these stabilization mechanisms, proteins from both animal and plant sources are being employed in many food-grade foams [95,96]. Alves et al., (2022) [97], evaluated the structural and foaming properties of mixing whey (WPI) and soy protein (SPI) isolates in different ratios before and after heat treatment. They found out that foam capacity (FC) values were similar for all of the samples despite their ratio and the submitted heat treatment. However, the blends of the two proteins negatively affected the foam stability (FS) even at moderate blends, with further antagonistic effect after heat treatment. They hypothesized that, even in small amounts, the more hydrated SP aggregates sterically prevented the formation of a strong and compact viscoelastic protein film at the air-water interface. Moreover, the high temperature contributed to the formation of insoluble aggregates, mainly stabilized by hydrophobic interactions, of both WPI and SP which further reduced the flexibility of the interfacial film. Both of these phenomena contributed to the reduction in FS values for the mixed proteins samples. Similar results were obtained by the study of Krentz et al., (2022) [98], where the authors evaluated the foaming properties of a mixture of casein micelles (CMs) and pea protein isolate (PPI). The blends were compared to skim milk and pea protein isolate slurries which, respectively, exhibited the highest and the lowest values for both FC and FS. The incorporation of CMs in the blends enhanced the foaming properties of PPI control, while PPI presence did not improve values of CMs control. It is then reasonable to say that in this study, pea protein aggregates behaved similarly to the soy protein aggregates, sterically preventing the formation of a strong viscoelastic film at the bubble interfaces and destabilizing the overall system.

Therefore, it can be concluded that, even though producers may be able to find a useful ratio of dairy and plant proteins to obtain a required functionality, their mix does not manifest a synergic effect in stabilizing a foam system; a property which is instead given by the sole characteristics of the proteins employed.

However, proteins techno-functionality can be modified using different and non-conventional physicochemical treatments in order to stimulate protein-protein interaction and improve the overall stability of the system. Up to date, there are not many studies regarding the possibility of treatment applications on dairy and plant proteins in mixed system to increase their colloidal properties. More knowledge of these systems and their possible manipulation is therefore required in the next years.

## 5. Possible Approaches to Improve Dairy-Plant Proteins Interaction and Techno-Functionalities

To try to overcome dairy and plant proteins limits in forming and stabilizing any different colloidal system, lately new approaches have been explored. For example, the design of innovative food products based on the combination of milk and plant proteins have also been attempted exploiting microorganisms. While microbial fermentation is well known to modify milk protein behavior, it has also been employed to reduce many off-flavors linked to beans presence as well to improve plant proteins techno-functional properties on different plant-based food systems [99,100]. Canon et al., (2022) [101], manufactured plant-based yogurt alternatives by emulsifying milk and lupin protein and fermenting it with a coculture of several species of lactic acid bacteria. The addition of the fermentation process presented encouraging results; indeed, some cocultures developed a more firm and viscous structure with a higher water holding capacity, in particular, when the milk/lupin protein ratio was 67:33. Moreover, these yogurt alternatives were sensorially discriminated on the sole protein ratio and fat type, not from the different starters employed. These findings could thus lead to a wide variety of formulations with several interesting features that could also promote the consumption of such innovative plant-based products. Additionally, the fermentation with different starting cultures could represent a strategic tool to manufacture newly mixed foods with the desired techno-functional and sensorial properties.

Other alternative technologies have also been studied recently to modify dairy and plant protein behavior. Pulsed electric field (PEF), for example, are being used for their ability to change the protein structure and, consequently, their physicochemical properties [102,103]. Indeed, PEF treatments can improve protein interactions by promoting the unfolding of the molecules and the polarization of the amino acids by exposing hydrophobic regions as well as sulfhydryl groups and, thus, enhancing protein aggregation. Several studies applied PEF treatments on dairy, and plant proteins and the main effects are summarized in Table 6.

The effectiveness of PEF technology to improve protein functionality highly depends on the specific conditions used (intensity, time, and temperature of the treatment), which could thus be tailored for each type of protein to obtain the desired features in every colloidal system. However, up to date, PEF has not yet been applied in a system where dairy and plant proteins coexist. Thus, more studies are needed to elucidate if this technology could promote their interaction and affinity.

High pressure processing (HPP) is a nonthermal processing technology used in the food industry to extend food products shelf-life [111], but it has been also employed to modify structure and functionality of food proteins in colloidal systems. While HPP for animal and dairy proteins have been largely studied, little knowledge exists nowadays on the effects of this processing method on alternative proteins. Queirós et al., (2018) [112], reviewed the recent applications on a large variety of plant proteins and concluded that HPP can tailor their functionalities by inducing unfolding paths and better exposition of functional groups promoting their aggregation, solubility, emulsifying, gelling, and foaming properties. Sim et al., (2020) [113], applied HPP treatment to develop plant protein (mung bean, chickpea, pea, lentil, and faba bean) gels and emulsions and compared the results to commercial dairy yogurts. The study revealed that HPP developed viscoelastic gels and emulsions with comparable gel strength and viscosities to the controls, proposing a new methodology to develop plant-based yogurt alternatives. However, how HPP affects techo-functionalities of the proteins highly depends on a complex series of relationships between the intrinsic characteristics and type of protein, the environmental conditions, and the high-pressure parameters. Thus, in this context, when a system is composed of two or more different biopolymers, such as dairy and plant proteins, the HPP conditions to improve properties of both are most likely to be incompatible, thus, if this technology is to be used to combine dairy and plant proteins in a mixture, perhaps it would be wise to treat them separately, tailoring specific characteristics and then proceed with their mixture.

Nowadays, many other innovative processes are being investigated to try to improve plant-based proteins’ physicochemical properties, such as partial hydrolysis, ohmic heating and freeze-thaw cycle, which are capable of modifying proteins’ structure and intermolecular forces [114,115,116]. However, currently, little is known about these modifications when milk and plant proteins are both present in the same medium. It will therefore be a task and trend for the future food industry research to investigate how these processes can affect a single or both proteins and if it could be useful to improve their interactions for the development of innovations in the food industry.

## 6. Sensory Attributes of Mixed Systems

An important feature of any food is its sensorial attributes; required consumer intent, desirable texture, taste, flavor, among others. However, studies regarding the sensory evaluation of mixed proteins systems are still scarce. Zare et al., (2011) [78], compared the smoothness, graininess, flavor, color, and overall acceptance of two supplemented yogurts. The replacement of skim milk powder with lentil flour was evaluated sensorially. The yogurt supplemented with 1.2 and 3% of lentil flour showed no significant difference in smoothness, graininess, flavor, and overall acceptance when compared to yogurts supplemented with 1.2 and 3% of skim milk powder. However, in the color parameter, yogurts added with 2 and 3% of lentil flour were different from 2 and 3% skim milk yogurt. Thus, the impact of the addition of vegetable protein was not perceived by the consumers in the concentration studied, indicating that lentil flour can be used to fortify yogurts without sensorial loss. The concentration of plant protein added in dairy products must be high enough to cause desirable changes in the functional properties and, at the same time, cause minimum interference in the sensorial attributes.

The sensorial impact caused by increasing concentration of pea protein in yogurts produced using several starter cultures was evaluated by Yousseef et al., (2016) [77]. As pea concentration increases, the intensity of the terms pea, earth, smoked, and vinegar increased, which are considered negative sensory characteristics, while the positive terms dairy and creamy decreased in intensity. Among the pea concentrations studied, yogurts containing 20 to 40% of pea protein were characterized as products with undesirable features, while 10% pea protein concentration was considered the closest to the control yogurt sample. In addition, the fermentation process also showed the potentiality of decreasing the undesirable beany flavor of mixed milk-pea gels [117]. However, the type of metabolites, as well as the microorganism growth, depend on the composition of the gel matrix [118]. Canon et al., (2022) [101], also manufactured mixed dairy and plant protein yogurt alternatives, mixing, in particular, skim milk powder or whey proteins with lupin protein isolate and milk fat or coconut oil. They evaluated not only the protein type proportion but also the fermentation with several lactic acid bacteria strains. The sensorial results obtained showed that yogurt alternatives were discriminated only on the basis of protein ratios and fat components but not of starters. In particular, the milk/lupin ratio of 67:33 was more accepted than the 50:50 ratio; however, the employment of cocultures of lactic acid bacteria produced different aroma compounds, which increased 50:50 acceptance. Thus, the sensory changes promoted by the addition of plant proteins cannot be underestimated, and studies regarding the maximum quantity of protein addition are highly required.

Lastly, Grygorczyk et al., (2013) [76], using napping methodology investigated the effect of the order of homogenization in the texture of systems formed by soy milk and cow’s milk. The homogenization of milk with cream in the presence or absence of soymilk leads to yogurts with high thickness, roughness, and mouthcoating. When the cream was homogenized in soymilk with posterior addition of skim milk, the formed gel exhibits thinner and watery features. The perception of fatty attributes was also influenced by the homogenization order, since the fat content of all samples was the same. The samples, where the aggregation of milk proteins started first, had more fatty-related attributes, while the opposite happened when the aggregation of the protein occurred at the same time, showing that, how the fat globules are disposed in the matrix, can influence the perception of fats in the product.

## 7. Conclusions and Perspectives

The studies address evidence of concept, indicating that combination of plant and milk proteins can be used to module colloidal systems with direct application in the food industry. Some studies pointed out that the addition of plant protein can solve some technological problems such as the production of high-protein dairy beverages which tend to form a gel in the packaging. Moreover, the presence of dairy proteins can increase the solubility of some plant proteins in mixed protein dispersions. However, plant protein solubility remains the greatest challenge to overcome in the designing and manufacturing of such products. The low solubility can in fact lead to complex intermolecular interactions between the proteins and the solvent, causing higher molecular aggregates and precipitates.

On the other hand, this complex behavior could be useful for the development of a gel system. Even though there is no general rule for the manufacturing of mixed dairy and plant proteins gels, knowing the specific techno-functional properties of all the protein fractions involved and how they are influenced by environmental conditions such as pH and temperature, may allow the development of innovative gel products such as yogurt, cheese analogues, and beverages characterized by appropriate textural and sensorial properties. Moreover, different pre-treatments could be applied to the proteins such as ultrasounds and enzymatic reactions that can improve their intermolecular interactions and impact the overall structure of the final gel.

Regarding the emulsion colloidal system, mixed systems of dairy and plant proteins manifested a better synergy for stabilizing the system. Indeed, the presence of both types of protein at the interfaces of the emulsions (*o*/*w* or *w*/*o*) stabilized the droplets for long periods of time and created a denser interfacial layer, inhibiting at the same time creaming and sedimentation. The results here presented could be of paramount importance for the design of enhanced emulsion food products such as salad dressings, sauces, and cheese analogues.

Finally, foam systems with the presence of both proteins were taken into consideration, reaching the conclusion that their mixture does not manifest a synergic effect in stabilizing a foam system. Nonetheless, both proteins were able to create a viscoelastic film around each air bubble, they did not interact with themself, and the stabilization mechanisms were only given via the characteristics of the single proteins employed, where dairy sources presented higher foaming properties than plant ones.

However, to try to enhance these colloidal systems and thus promote their application in the food industry, emergent and innovative approaches are being evaluated to modify the techno-functional properties of both dairy and plant proteins. For example, precise fermentation, pulsed electric field, and high hydrostatic pressure are able to modify protein structures and physicochemical properties and can therefore be employed in these binary systems to obtain the desired characteristics and promote their application in the food system. Moreover, the sensorial profile of such foods needs always to be taken into account since it can also be responsible for consumer rejection. In this context, a balanced proportion of milk and plant proteins needs to be achieved depending on the desired characteristics of the final product.

Thus, new studies are needed in the near future to evaluate the applicability of pre-treatments to promote dairy and plant proteins’ coexistence, to improve their functional and sensorial properties, and, consequently, to open a new market category of innovative food products.

## Figures and Tables

**Table 1:** Components of raw milk.

Component	Proportion (%)
Water	85–87
Lipids	3.8–5.5
Lactose	4.8–5.0
Proteins	2.9–3.5

**Table 2:** Plant proteins and their functionalities based on native physicochemical properties.

Plant Source	Physicochemical Properties	Functionality
Soy, almond, rice	Hydrophilicity; surface charge; hydrogen bonding	Solubility
Soy, pea, lentils, beans	Aggregative behavior after thermal and pH denaturation; electrostatic and hydrophobic interactions; disulfide bonding	Gelling
Soy, pea, faba, sunflower, pumpkin	Surface tension; interfacial film forming ability; amphipathic behavior	Emulsifying
Potato, pea, lentils, chickpea	Surface tension; interfacial film forming ability; amphipathic behavior	Foaming

**Table 3:** Summary of the key results obtained for mixed protein dispersions.

Mixed Dispersions	Functionalities	Reference
Milk/Pea	Antagonistic effect on protein solubility	Ben-Harb et al., (2018) [18]
Whey/Rice	Improved solubility with pH shift	Wang et al., (2019) [52]
Caseinates/Rice	Improved solubility with pH shift	Wang et al., (2018) [53]
Milk/Soy	Higher viscosities of the dispersions with decreased coagulation	Singh et al., (2019) [54]

**Table 4:** Summary of mixed dairy-plant gel.

Mixed Heat-Induced Gel	Functionalities	Reference
Caseins/Pea/soy	No interaction in the gel formation and presence of distinct phases	Silva et. al., 2018 [66]
Whey/Pea	Increased gelation temperature; modulation of gel structure and rheological properties; increased gel stiffness; increased gel homogeneity at isoelectric pH	Wong et. al., 2013 [64]
Whey/Soy	Formation of aggregates with disulfide bonds; reduced gel strength	Corredig et. al., 2015 [87]
Mixed Acid-induced gel		
Whey/Pea	Decreased gelation pH; decreased gel stiffness; no interactions	Chihi et. al., 2018 [63]
Milk/Pea	Faster gelation; increased acidity; increased syneresis; decreased gel firmness	Yousseef et. al., 2016 [77]
Milk/Lentil	Similar syneresis and rheological behavior to milk control	Zare et. al., 2011 [78]
Enzymatic gel		
Whey/Soy	Increased gel hardness; optimal water holding capacity; decreased rheological properties	Cui et. al., 2020 [80]

**Table 5:** Summary of mixed dairy: plant emulsions.

Mixed Emulsions	Functionalities	Reference
Caseins/Soy	Improved stability; small droplet sizes	Ji et. al., 2015 [90]
Milk/Pea	High stability; small droplet sizes; dense interfacial layer	Hinderink et. al., 2019 [17]
Milk/Pea/soy	Reduced droplet sizes and improved heat-stability	Liang et. al., 2016 [91]
Milk/Faba	Overall optimal stability; decreased solubility; larger particle sizes	Le Roux et. al., 2020 [92]
Caseins/Soy	Enhanced encapsulation ability for vitamin a and lycopene	Ho et. al., 2018 [93]

**Table 6:** Adapted with permission from Taha et al., (2022) [102].

Protein	Effects on Functionality	References
Whey Protein Isolate (WPI)	Improved gelling properties of WPI when treated with an intensity lower than 45 kV/cm. However, weaker gel strengths compared to heat-treated gels.	Jin et al., 2013; Rodrigues et al., 2015 [104,105]
Caseins and WPI	Increased rate of unfolding proteins and their surface hydrophobicity	Sharma et al., 2016; Subasi et al., 2021 [106,107]
Soy protein isolate (SPI)	Decreased solubility and surface hydrophobicity	Li et al., 2007 [108]
Canola protein	Improved solubility, foaming and emulsifying properties	Zhang et al., 2017 [109]
Sunflower protein	Reduced interfacial tension at protein/water interface	Subasi et al., 2021 [107]
Pea protein isolate (PPI)	Increased surface hydrophobicity and gelling properties	Chen et al., 2022 [110]

## Data Availability

No new data were created for the production of this manuscript. All of the data here discussed and presented are available in the relative references here cited and listed.

## References

[1] Poore J., Nemecek T. (2018). Reducing Food’s Environmental Impacts through Producers and Consumers. Science.

[2] Clune S., Crossin E., Verghese K. (2017). Systematic Review of Greenhouse Gas Emissions for Different Fresh Food Categories. J. Clean. Prod..

[3] Matsumiya K., Murray B.S. (2016). Soybean Protein Isolate Gel Particles as Foaming and Emulsifying Agents. Food Hydrocoll..

[4] Bergsma J. (2017). Vegan Cheese Analogue.

[5] Lipan L., Rusu B., Sendra E., Hernández F., Vázquez-Araújo L., Vodnar D.C., Carbonell-Barrachina Á.A. (2020). Spray Drying and Storage of Probiotic-Enriched Almond Milk: Probiotic Survival and Physicochemical Properties. J. Sci. Food Agric..

[6] Schreuders F.K.G., Dekkers B.L., Bodnár I., Erni P., Boom R.M., van der Goot A.J. (2019). Comparing Structuring Potential of Pea and Soy Protein with Gluten for Meat Analogue Preparation. J. Food Eng..

[7] Lonnie M., Johnstone A.M. (2020). The Public Health Rationale for Promoting Plant Protein as an Important Part of a Sustainable and Healthy Diet. Nutr. Bull..

[8] Budhathoki S., Sawada N., Iwasaki M., Yamaji T., Goto A., Kotemori A., Ishihara J., Takachi R., Charvat H., Mizoue T. (2019). Association of Animal and Plant Protein Intake with All-Cause and Cause-Specific Mortality in a Japanese Cohort. JAMA Intern. Med..

[9] Qian F., Liu G., Hu F.B., Bhupathiraju S.N., Sun Q. (2019). Association between Plant-Based Dietary Patterns and Risk of Type 2 Diabetes: A Systematic Review and Meta-Analysis. JAMA Intern. Med..

[10] Sá A.G.A., Moreno Y.M.F., Carciofi B.A.M. (2020). Plant Proteins as High-Quality Nutritional Source for Human Diet. Trends Food Sci. Technol..

[11] Nikbakht Nasrabadi M., Sedaghat Doost A., Mezzenga R. (2021). Modification Approaches of Plant-Based Proteins to Improve Their Techno-Functionality and Use in Food Products. Food Hydrocoll..

[12] Goulding D.A., Fox P.F., O’Mahony J.A. (2019). Milk Proteins: An Overview. Milk Proteins: From Expression to Food.

[13] Uluko H., Liu L., Lv J.P., Zhang S.W. (2016). Functional Characteristics of Milk Protein Concentrates and Their Modification. Crit. Rev. Food Sci. Nutr..

[14] Pouliot Y. (2008). Membrane Processes in Dairy Technology-From a Simple Idea to Worldwide Panacea. Int. Dairy J..

[15] Schmitt C., Bovetto L., Buczkowski J., De Oliveira Reis G., Pibarot P., Amagliani L., Dombrowski J. (2021). Plant Proteins and Their Colloidal State. Curr. Opin. Colloid Interface Sci..

[16] Guyomarc’h F., Arvisenet G., Bouhallab S., Canon F., Deutsch S.M., Drigon V., Dupont D., Famelart M.H., Garric G., Guédon E. (2021). Mixing Milk, Egg and Plant Resources to Obtain Safe and Tasty Foods with Environmental and Health Benefits. Trends Food Sci. Technol..

[17] Hinderink E.B.A., Münch K., Sagis L., Schroën K., Berton-Carabin C.C. (2019). Synergistic Stabilisation of Emulsions by Blends of Dairy and Soluble Pea Proteins: Contribution of the Interfacial Composition. Food Hydrocoll..

[18] Ben-Harb S., Panouillé M., Huc-Mathis D., Moulin G., Saint-Eve A., Irlinger F., Bonnarme P., Michon C., Souchon I. (2018). The Rheological and Microstructural Properties of Pea, Milk, Mixed Pea/Milk Gels and Gelled Emulsions Designed by Thermal, Acid, and Enzyme Treatments. Food Hydrocoll..

[19] Foroutan A., Guo A.C., Vazquez-Fresno R., Lipfert M., Zhang L., Zheng J., Badran H., Budinski Z., Mandal R., Ametaj B.N. (2019). Chemical Composition of Commercial Cow’s Milk. J. Agric. Food Chem..

[20] Walstra P., Wouters J.T.M., Geurts T.J. (2006). Dairy Science and Technology.

[21] Horne D.S. (2019). Casein Micelle Structure and Stability. Milk Proteins: From Expression to Food.

[22] Edwards P.J.B., Jameson G.B. (2019). Structure and Stability of Whey Proteins. Milk Proteins: From Expression to Food.

[23] Holt C., Carver J.A., Ecroyd H., Thorn D.C. (2013). Invited Review: Caseins and the Casein Micelle: Their Biological Functions, Structures, and Behavior in Foods1. J. Dairy Sci..

[24] Carter B.G., Cheng N., Kapoor R., Meletharayil G.H., Drake M.A. (2021). Invited Review: Microfiltration-Derived Casein and Whey Proteins from Milk. J. Dairy Sci..

[25] Carr A., Golding M. (2016). Functional Milk Proteins Production and Utilization: Casein-Based Ingredients. Advanced Dairy Chemistry: Volume 1B: Proteins: Applied Aspects.

[26] Huppertz T., Fox P.F., Kelly A.L. (2017). The Caseins: Structure, Stability, and Functionality. Proteins in Food Processing.

[27] Huppertz T., Gazi I., Luyten H., Nieuwenhuijse H., Alting A., Schokker E. (2017). Hydration of Casein Micelles and Caseinates: Implications for Casein Micelle Structure. Int. Dairy J..

[28] Hammam A.R.A., Martínez-Monteagudo S.I., Metzger L.E. (2021). Progress in Micellar Casein Concentrate: Production and Applications. Compr. Rev. Food Sci. Food Saf..

[29] Oliveira I.C., de Paula Ferreira I.E., Casanova F., Cavallieri A.L.F., Lima Nascimento L.G., de Carvalho A.F., Nogueira Silva N.F. (2022). Colloidal and Acid Gelling Properties of Mixed Milk and Pea Protein Suspensions. Foods.

[30] Smithers G.W. (2008). Whey and Whey Proteins-From “Gutter-to-Gold”. Int. Dairy J..

[31] Farrell H.M., Jimenez-Flores R., Bleck G.T., Brown E.M., Butler J.E., Creamer L.K., Hicks C.L., Hollar C.M., Ng-Kwai-Hang K.F., Swaisgood H.E. (2004). Nomenclature of the Proteins of Cows’ Milk-Sixth Revision. J. Dairy Sci..

[32] Guyomarc’h F., Famelart M.H., Henry G., Gulzar M., Leonil J., Hamon P., Bouhallab S., Croguennec T. (2015). Current Ways to Modify the Structure of Whey Proteins for Specific Functionalities—A Review. Dairy Sci. Technol..

[33] Loveday S.M. (2020). Plant Protein Ingredients with Food Functionality Potential. Nutr. Bull..

[34] Tan M., Nawaz M.A., Buckow R. (2021). Functional and Food Application of Plant Proteins–A Review. Food Rev. Int..

[35] Bessada S.M.F., Barreira J.C.M., Oliveira M.B.P.P. (2019). Pulses and Food Security: Dietary Protein, Digestibility, Bioactive and Functional Properties. Trends Food Sci. Technol..

[36] Hara-Nishimura I., Shimada T., Hatano K., Takeuchi Y., Nishimura M. (1998). Transport of Storage Proteins to Protein Storage Vacuoles Is Mediated by Large Precursor-Accumulating Vesicles. Plant Cell.

[37] Boye J., Zare F., Pletch A. (2010). Pulse Proteins: Processing, Characterization, Functional Properties and Applications in Food and Feed. Food Res. Int..

[38] Gumus C.E., Decker E.A., McClements D.J. (2017). Impact of Legume Protein Type and Location on Lipid Oxidation in Fish Oil-in-Water Emulsions: Lentil, Pea, and Faba Bean Proteins. Food Res. Int..

[39] Aachary A.A., Thiyam-Hollander U., Eskin M.N.A. (2014). Canola/Rapeseed Proteins and Peptides. Applied Food Protein Chemistry.

[40] Loveday S.M. (2019). Food Proteins: Technological, Nutritional, and Sustainability Attributes of Traditional and Emerging Proteins. Annu. Rev. Food Sci. Technol..

[41] Mohammed K., Obadi M., Omedi J.O., Letsididi K.S., Koko M., Zaaboul F., Siddeeg A., Liu Y. (2018). Effect of Sunflower Meal Protein Isolate (SMPI) Addition on Wheat Bread Quality. J. Acad. Ind. Res..

[42] Pereira J., Zhou G., Zhang W. (2020). Effects of Rice Flour on Emulsion Stability, Organoleptic Characteristics and Thermal Rheology of Emulsified Sausage. J. Food Nutr. Res..

[43] Akharume F.U., Aluko R.E., Adedeji A.A. (2021). Modification of Plant Proteins for Improved Functionality: A Review. Compr. Rev. Food Sci. Food Saf..

[44] Nwachukwu I.D., Aluko R.E. (2021). Chapter 1: Food Protein Structures, Functionality and Product Development. Food Chemistry, Function and Analysis.

[45] Roberts C.J. (2014). Protein Aggregation and Its Impact on Product Quality. Curr. Opin. Biotechnol..

[46] Nicolai T., Chassenieux C. (2019). Heat-Induced Gelation of Plant Globulins. Curr. Opin. Food Sci..

[47] Foegeding E.A. (2015). Food Protein Functionality-A New Model. J. Food Sci..

[48] Milani J.M., Golkar A. (2019). Introductory Chapter: Some New Aspects of Colloidal Systems in Foods. Some New Aspects of Colloidal Systems in Foods.

[49] Alrosan M., Tan T.C., Easa A.M., Gammoh S., Alu’datt M.H. (2022). Molecular Forces Governing Protein-Protein Interaction: Structure-Function Relationship of Complexes Protein in the Food Industry. Crit. Rev. Food Sci. Nutr..

[50] Pace C.N., Treviño S., Prabhakaran E., Scholtz J.M., Franks F., Wilson K., Daniel R.M., Halling P.J., Clark D.S., Purkiss A. (2004). Protein Structure, Stability and Solubility in Water and Other Solvents. Philos. Trans. R. Soc. B Biol. Sci..

[51] Shire S.J., Shahrokh Z., Liu J. (2004). Challenges in the Development of High Protein Concentration Formulations. J. Pharm. Sci..

[52] Wang R., Xu P., Chen Z., Zhou X., Wang T. (2019). Complexation of Rice Proteins and Whey Protein Isolates by Structural Interactions to Prepare Soluble Protein Composites. LWT.

[53] Wang T., Yue M., Xu P., Wang R., Chen Z. (2018). Toward Water-Solvation of Rice Proteins via Backbone Hybridization by Casein. Food Chem..

[54] Singh J., Prakash S., Bhandari B., Bansal N. (2019). Ultra High Temperature (UHT) Stability of Casein-Whey Protein Mixtures at High Protein Content: Heat Induced Protein Interactions. Food Res. Int..

[55] Yousefi N., Abbasi S. (2022). Food Proteins: Solubility & Thermal Stability Improvement Techniques. Food Chem. Adv..

[56] Saricaoglu F.T. (2020). Application of High-Pressure Homogenization (HPH) to Modify Functional, Structural and Rheological Properties of Lentil (Lens Culinaris) Proteins. Int. J. Biol. Macromol..

[57] Wang F., Zhang Y., Xu L., Ma H. (2020). An Efficient Ultrasound-Assisted Extraction Method of Pea Protein and Its Effect on Protein Functional Properties and Biological Activities. LWT.

[58] Sánchez-Reséndiz A., Rodríguez-Barrientos S., Rodríguez-Rodríguez J., Barba-Dávila B., Serna-Saldívar S.O., Chuck-Hernández C. (2018). Phosphoesterification of Soybean and Peanut Proteins with Sodium Trimetaphosphate (STMP): Changes in Structure to Improve Functionality for Food Applications. Food Chem..

[59] Eckert E., Han J., Swallow K., Tian Z., Jarpa-Parra M., Chen L. (2019). Effects of Enzymatic Hydrolysis and Ultrafiltration on Physicochemical and Functional Properties of Faba Bean Protein. Cereal Chem..

[60] Yildiz G., Ding J., Andrade J., Engeseth N.J., Feng H. (2018). Effect of Plant Protein-Polysaccharide Complexes Produced by Mano-Thermo-Sonication and PH-Shifting on the Structure and Stability of Oil-in-Water Emulsions. Innov. Food Sci. Emerg. Technol..

[61] Kontogeorgis G.M., Kiil S. (2016). Introduction to Colloid and Surface Chemistry. Introduction to Applied Colloid and Surface Chemistry.

[62] Gunasekaran S., Ak M.M. (2000). Dynamic Oscillatory Shear Testing of Foods-Selected Applications. Trends Food Sci. Technol..

[63] Chihi M.L., Sok N., Saurel R. (2018). Acid Gelation of Mixed Thermal Aggregates of Pea Globulins and β-Lactoglobulin. Food Hydrocoll..

[64] Wong D., Vasanthan T., Ozimek L. (2013). Synergistic Enhancement in the Co-Gelation of Salt-Soluble Pea Proteins and Whey Proteins. Food Chem..

[65] Silva J.V.C., Balakrishnan G., Schmitt C., Chassenieux C., Nicolai T. (2018). Heat-Induced Gelation of Aqueous Micellar Casein Suspensions as Affected by Globular Protein Addition. Food Hydrocoll..

[66] Silva J.V.C., Cochereau R., Schmitt C., Chassenieux C., Nicolai T. (2019). Heat-Induced Gelation of Mixtures of Micellar Caseins and Plant Proteins in Aqueous Solution. Food Res. Int..

[67] Silva J.V.C., Jacquette B., Amagliani L., Schmitt C., Nicolai T., Chassenieux C. (2019). Heat-Induced Gelation of Micellar Casein/Plant Protein Oil-in-Water Emulsions. Colloids Surf. A Physicochem. Eng. Asp..

[68] Mession J.L., Roustel S., Saurel R. (2017). Interactions in Casein Micelle–Pea Protein System (Part I): Heat-Induced Denaturation and Aggregation. Food Hydrocoll..

[69] Nicolai T., Chassenieux C. (2021). Heat-Induced Gelation of Casein Micelles. Food Hydrocoll..

[70] Huppertz T., Nieuwenhuijse H. (2022). Constituent Fouling during Heat Treatment of Milk: A Review. Int. Dairy J..

[71] Chihi M.L., Mession J.L., Sok N., Saurel R. (2016). Heat-Induced Soluble Protein Aggregates from Mixed Pea Globulins and β-Lactoglobulin. J. Agric. Food Chem..

[72] Roesch R.R., Corredig M. (2005). Heat-Induced Soy-Whey Proteins Interactions: Formation of Soluble and Insoluble Protein Complexes. J. Agric. Food Chem..

[73] McCann T.H., Guyon L., Fischer P., Day L. (2018). Rheological Properties and Microstructure of Soy-Whey Protein. Food Hydrocoll..

[74] Totosaus A., Montejano J.G., Salazar J.A., Guerrero I. (2002). A Review of Physical and Chemical Protein-Gel Induction. Int. J. Food Sci. Technol..

[75] Lucey J.A. (2020). Milk Protein Gels. Milk Proteins: From Expression to Food.

[76] Grygorczyk A., Alexander M., Corredig M. (2013). Combined Acid- and Rennet-Induced Gelation of a Mixed Soya Milk-Cow’s Milk System. Int. J. Food Sci. Technol..

[77] Yousseef M., Lafarge C., Valentin D., Lubbers S., Husson F. (2016). Fermentation of Cow Milk and/or Pea Milk Mixtures by Different Starter Cultures: Physico-Chemical and Sensorial Properties. LWT.

[78] Zare F., Boye J.I., Orsat V., Champagne C., Simpson B.K. (2011). Microbial, Physical and Sensory Properties of Yogurt Supplemented with Lentil Flour. Food Res. Int..

[79] Martin A.H., De Los Reyes Jiménez M.L., Pouvreau L. (2016). Modulating the Aggregation Behaviour to Restore the Mechanical Response of Acid Induced Mixed Gels of Sodium Caseinate and Soy Proteins. Food Hydrocoll..

[80] Cui Q., Wang G., Gao D., Wang L., Zhang A., Wang X., Xu N., Jiang L. (2020). Improving the Gel Properties of Transgenic Microbial Transglutaminase Cross-Linked Soybean-Whey Mixed Protein by Ultrasonic Pretreatment. Process Biochem..

[81] Ma Z., Li L., Wu C., Huang Y., Teng F., Li Y. (2022). Effects of Combined Enzymatic and Ultrasonic Treatments on the Structure and Gel Properties of Soybean Protein Isolate. LWT.

[82] Means A.K., Grunlan M.A. (2019). Modern Strategies to Achieve Tissue-Mimetic, Mechanically Robust Hydrogels. ACS Macro Lett..

[83] Jansens K.J.A., Rombouts I., Grootaert C., Brijs K., Van Camp J., Van der Meeren P., Rousseau F., Schymkowitz J., Delcour J.A. (2019). Rational Design of Amyloid-Like Fibrillary Structures for Tailoring Food Protein Techno-Functionality and Their Potential Health Implications. Compr. Rev. Food Sci. Food Saf..

[84] Khalesi H., Sun C., He J., Lu W., Fang Y. (2021). The Role of Amyloid Fibrils in the Modification of Whey Protein Isolate Gels with the Form of Stranded and Particulate Microstructures. Food Res. Int..

[85] Wu H., Nian Y., Liu Y., Zhang Y., Hu B. (2022). Formation of Pea Protein Amyloid Fibrils to Stabilize High Internal Phase Emulsions for Encapsulation of Lutein. J. Funct. Foods.

[86] Ge J., Sun C., Chang Y., Sun M., Zhang Y., Fang Y. (2022). Heat-Induced Pea Protein Isolate Gels Reinforced by Panda Bean Protein Amyloid Fibrils: Gelling Properties and Formation Mechanism. Food Res. Int..

[87] Grygorczyk A., Duizer L., Lesschaeve I., Corredig M. (2014). Gelation of Recombined Soymilk and Cow’s Milk Gels: Effect Ofhomogenization Order and Mode of Gelation on Microstructure Andtexture of the Final Matrix. Food Hydrocoll..

[88] Derkach S.R. (2009). Rheology of Emulsions. Adv. Colloid Interface Sci..

[89] Singh H., Gallier S. (2017). Nature’s Complex Emulsion: The Fat Globules of Milk. Food Hydrocoll..

[90] Ji J., Zhang J., Chen J., Wang Y., Dong N., Hu C., Chen H., Li G., Pan X., Wu C. (2015). Preparation and Stabilization of Emulsions Stabilized by Mixed Sodium Caseinate and Soy Protein Isolate. Food Hydrocoll..

[91] Liang Y., Wong S.S., Pham S.Q., Tan J.J. (2016). Effects of Globular Protein Type and Concentration on the Physical Properties and Flow Behaviors of Oil-in-Water Emulsions Stabilized by Micellar Casein-Globular Protein Mixtures. Food Hydrocoll..

[92] Le Roux L., Mejean S., Chacon R., Lopez C., Dupont D., Deglaire A., Nau F., Jeantet R. (2020). Plant Proteins Partially Replacing Dairy Proteins Greatly Influence Infant Formula Functionalities. LWT.

[93] Ho K.K.H.Y., Schroën K., San Martín-González M.F., Berton-Carabin C.C. (2018). Synergistic and Antagonistic Effects of Plant and Dairy Protein Blends on the Physicochemical Stability of Lycopene-Loaded Emulsions. Food Hydrocoll..

[94] Patel S.G., Siddaiah M. (2018). Formulation and Evaluation of Effervescent Tablets: A Review. J. Drug Deliv. Ther..

[95] Mohanan A., Nickerson M.T., Ghosh S. (2020). Utilization of Pulse Protein-Xanthan Gum Complexes for Foam Stabilization: The Effect of Protein Concentrate and Isolate at Various PH. Food Chem..

[96] Odelli D., Sarigiannidou K., Soliani A., Marie R., Mohammadifar M.A., Jessen F., Spigno G., Vall-llosera M., De Carvalho A.F., Verni M. (2022). Interaction between Fish Skin Gelatin and Pea Protein at Air-Water Interface after Ultrasound Treatment. Foods.

[97] Alves A.C., Martha L., Casanova F., Tavares G.M. (2022). Structural and Foaming Properties of Whey and Soy Protein Isolates in Mixed Systems before and after Heat Treatment. Food Sci. Technol. Int..

[98] Krentz A., García-Cano I., Jiménez-Flores R. (2022). Functional, Textural, and Rheological Properties of Mixed Casein Micelle and Pea Protein Isolate Co-Dispersions. JDS Commun..

[99] Fischer E., Cayot N., Cachon R. (2022). Potential of Microorganisms to Decrease the “Beany” Off-Flavor: A Review. J. Agric. Food Chem..

[100] Clark A.J., Soni B.K., Sharkey B., Acree T., Lavin E., Bailey H.M., Stein H.H., Han A., Elie M., Nadal M. (2022). Shiitake Mycelium Fermentation Improves Digestibility, Nutritional Value, Flavor and Functionality of Plant Proteins. LWT.

[101] Canon F., Maillard M.B., Famelart M.H., Thierry A., Gagnaire V. (2022). Mixed Dairy and Plant-Based Yogurt Alternatives: Improving Their Physical and Sensorial Properties through Formulation and Lactic Acid Bacteria Cocultures. Curr. Res. Food Sci..

[102] Taha A., Casanova F., Šimonis P., Stankevič V., Gomaa M.A.E., Stirkė A. (2022). Pulsed Electric Field: Fundamentals and Effects on the Structural and Techno-Functional Properties of Dairy and Plant Proteins. Foods.

[103] Giteru S.G., Oey I., Ali M.A. (2018). Feasibility of Using Pulsed Electric Fields to Modify Biomacromolecules: A Review. Trends Food Sci. Technol..

[104] Jin S., Yin Y., Wang Y. (2013). Effects of Combined Pulsed Electric Field and Heat Treatment on Texture Characteristics of Whey Protein Gels. Nongye Jixie Xuebao/Trans. Chin. Soc. Agric. Mach..

[105] Rodrigues R.M., Martins A.J., Ramos O.L., Malcata F.X., Teixeira J.A., Vicente A.A., Pereira R.N. (2015). Influence of Moderate Electric Fields on Gelation of Whey Protein Isolate. Food Hydrocoll..

[106] Sharma P., Oey I., Everett D.W. (2016). Thermal Properties of Milk Fat, Xanthine Oxidase, Caseins and Whey Proteins in Pulsed Electric Field-Treated Bovine Whole Milk. Food Chem..

[107] Subaşı B.G., Jahromi M., Casanova F., Capanoglu E., Ajalloueian F., Mohammadifar M.A. (2021). Effect of Moderate Electric Field on Structural and Thermo-Physical Properties of Sunflower Protein and Sodium Caseinate. Innov. Food Sci. Emerg. Technol..

[108] Li Y., Chen Z., Mo H. (2007). Effects of Pulsed Electric Fields on Physicochemical Properties of Soybean Protein Isolates. LWT.

[109] Zhang L., Wang L.J., Jiang W., Qian J.Y. (2017). Effect of Pulsed Electric Field on Functional and Structural Properties of Canola Protein by Pretreating Seeds to Elevate Oil Yield. LWT.

[110] Chen Y., Wang T., Zhang Y., Yang X., Du J., Yu D., Xie F. (2022). Effect of Moderate Electric Fields on the Structural and Gelation Properties of Pea Protein Isolate. Innov. Food Sci. Emerg. Technol..

[111] Balasubramaniam V.M., Barbosa-Canovas G.V., Lelieveld H.L. (2016). High Pressure Processing of Food: Principles, Technology and Application.

[112] Queirós R.P., Saraiva J.A., da Silva J.A.L. (2018). Tailoring Structure and Technological Properties of Plant Proteins Using High Hydrostatic Pressure. Crit. Rev. Food Sci. Nutr..

[113] Sim S.Y.J., Hua X.Y., Henry C.J. (2020). A Novel Approach to Structure Plant-Based Yogurts Using High Pressure Processing. Foods.

[114] Kumar P.K., Sivabalan S., Parhi A., Sablani S.S. (2022). Modification of Pea Protein Isolate Functionality by Freeze–Thaw Cycling. J. Food Meas. Charact..

[115] Li X., Ye C., Tian Y., Pan S., Wang L. (2018). Effect of Ohmic Heating on Fundamental Properties of Protein in Soybean Milk. J. Food Process Eng..

[116] Sarigiannidou K., Odelli D., Jessen F., Mohammadifar M.A., Ajalloueian F., Vall-llosera M., Carvalho A.F., Casanova F. (2022). Interfacial Properties of Pea Protein Hydrolysate: The Effect of Ionic Strength. SSRN Electron. J..

[117] Pua A., Tang V.C.Y., Goh R.M.V., Sun J., Lassabliere B., Liu S.Q. (2022). Ingredients, Processing, and Fermentation: Addressing the Organoleptic Boundaries of Plant-Based Dairy Analogues. Foods.

[118] Harper A.R., Dobson R.C.J., Morris V.K., Moggré G.J. (2022). Fermentation of Plant-Based Dairy Alternatives by Lactic Acid Bacteria. Microb. Biotechnol..

